# Spatial and Pregnancy-Related Changes in the Protein, Amino Acid, and Carbohydrate Composition of Bovine Oviduct Fluid

**DOI:** 10.3390/ijms21051681

**Published:** 2020-02-29

**Authors:** Beatriz Rodríguez-Alonso, Veronica Maillo, Omar Salvador Acuña, Rebeca López-Úbeda, Alejandro Torrecillas, Constantine A. Simintiras, Roger Sturmey, Manuel Avilés, Patrick Lonergan, Dimitrios Rizos

**Affiliations:** 1 Department of Animal Reproduction, National Institute for Agricultural and Food Research and Technology (INIA), Ctra. de la Coruña KM 5.9, 28040 Madrid, Spain; rodriguezalonso.b@gmail.com (B.R.-A.); veronicamase@gmail.com (V.M.); 2 School of Agriculture and Food Science, University College Dublin, Belfield, Dublin 4, Ireland; c.simintiras@missouri.edu (C.A.S.); pat.lonergan@ucd.ie (P.L.); 3 Department of Cell Biology and Histology, Faculty of Medicine, Instituto Murciano de Investigación Biosanitaria (IMIB-Arrixaca), University of Murcia, 30100 Murcia, Spain; osacunam@uas.edu.mx (O.S.A.); rebeca.lopez2@um.es (R.L.-Ú.); maviles@um.es (M.A.); 4 Faculty of Veterinary and Zootechnics, Autonomous University of Sinaloa, Culiacan 80246, Mexico; 5 Department of Research, Animal Reproduction Biotechnology (ARBiotech), Culiacan 80015, Mexico; 6 Molecular Biology Section, SAI, University of Murcia, 30100 Murcia, Spain; alexts@um.es; 7 Center for Atherothrombosis and Metabolic Disease, Hull York Medical School, University of Hull, Hull HU3 2JZ, UK; Roger.Sturmey@hyms.ac.uk

**Keywords:** oviduct fluid, pregnancy, proteome, metabolome, bovine

## Abstract

Knowledge of how the biochemical composition of the bovine oviduct is altered due to the oviduct anatomy or the presence of an embryo is lacking. Thus, the aim of this study was to assess the effect of (І) oviduct anatomy and (ІІ) embryo presence on oviductal fluid (OF) protein, amino acid, and carbohydrate composition. Cross-bred beef heifers (*n* = 19) were synchronized and those in standing estrus were randomly allocated to a cyclic (non-bred) or pregnant (artificially inseminated) group. All heifers were slaughtered on Day 3 after estrus. The oviducts ipsilateral to the corpus luteum from each animal were isolated, straightened and cut, separating ampulla and isthmus. Each portion was flushed with 500 µl of PBS enabling recovery of the oocyte/embryo. Recovered unfertilized oocytes (cyclic group) and embryos (8-cell embryos; pregnant group) were located in the isthmus of the oviduct. Samples of flushing medium from the isthmus and ampulla were used for proteomic (*n* = 2 per group), amino acid (*n* = 5), and carbohydrate (*n* = 5) analysis. For proteomic analysis, total protein from cyclic and pregnant samples were labelled with different cyanine fluorescent probes and separated according to the isoelectric point using immobilized pH gradient strips (pH 3–10, 17 cm, Protean^®^ IEF cell system, Bio Rad). Second dimension was performed in a polyacrylamide gel (12%) in the presence of SDS using a Protean II XL system (Bio Rad). Images were obtained with a Typhoon 9410 scanner and analyzed with Progenesis SameSpots software v 4.0. Amino acid content in the OF was determined by high performance liquid chromatography (HPLC). Glucose, lactate, and pyruvate were quantified using microfluorometric enzyme-linked assays. For the proteomic assessment, the results of the image analysis were compared by ANOVA. For both amino acid and carbohydrate analyses, statistical analysis was carried out by 2-way ANOVA with the Holm-Sidak nonparametric post hoc analysis. On Day 3 post-estrus, OF composition varied based on (І) anatomical region, where isthmic metabolites were present in lower (i.e., lactate, glycine, and alanine) or higher (i.e., arginine) concentrations compared to the ampulla; and (ІІ) embryo presence, which was correlated with greater, arginine, phosphoglycerate kinase 1, serum albumin, α-1-antiproteinase and IGL@ protein concentrations. In conclusion, data indicate that the composition of bovine OF is anatomically dynamic and influenced by the presence of an early embryo.

## 1. Introduction

The oviduct is a tubular seromuscular organ connecting the ovary to the uterus providing a physiological environment for gamete interaction, fertilization, and early embryo development. During the first four days of embryo development, which occurs within the oviduct [1], embryonic nutritional requirements are presumed to be fulfilled largely by oviduct fluid (OF)—a complex mixture of proteins, amino acids, carbohydrates, ions, hormones, and extracellular vesicles, amongst other constituents [2]. OF composition is regulated by (І) selective transudate from the vasculature, and (ІІ) the secretory cells within the oviduct epithelial monolayer [3]. Moreover, OF composition is influenced spatially by the anatomical region of the oviduct [4,5,6] and temporally by the estrous cycle [7,8,9,10,11]. These spatiotemporal variations aid to shape the oviduct microenvironment in order to meet the needs of the gametes and zygote and to facilitate early embryo development [12]. Furthermore, OF is responsive to additional parameters such as the presence of gametes [13,14,15] and embryos [16].

Previous work from our group has identified regional differences in bovine oviduct epithelial cell (BOEC) gene expression (i.e., ampulla vs. isthmus; [17]) as well as differences induced by the presence of embryos [18]. However, the extent to which variations in transcript abundance translate to variations in OF composition is unclear. More specifically, variable correlations have been observed between BOEC mRNA abundance and OF protein levels [19,20]. Moreover, data on bovine OF proteomic and metabolic variations by oviduct region or due to the presence of an embryo are scarce, in contrast to the uterus where this relationship has been well described [21,22]. Based on the above, we hypothesized that the OF composition varies based on the anatomical region and the presence of an embryo. Thus, the aim of this study was to assess the effect of (І) oviduct anatomy and (ІІ) embryo presence on OF protein, amino acid, and carbohydrate composition. Furthermore, these data provide a unique opportunity to correlate existing oviductal epithelia cell gene expression profiles with OF composition during early embryo development.

## 2. Results

### 2.1. Sample Collection

**Figure 1:**
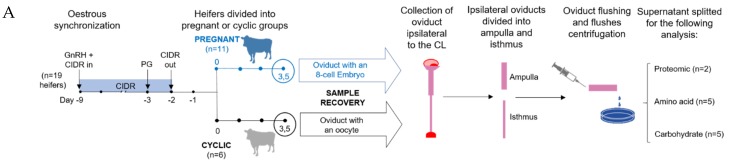

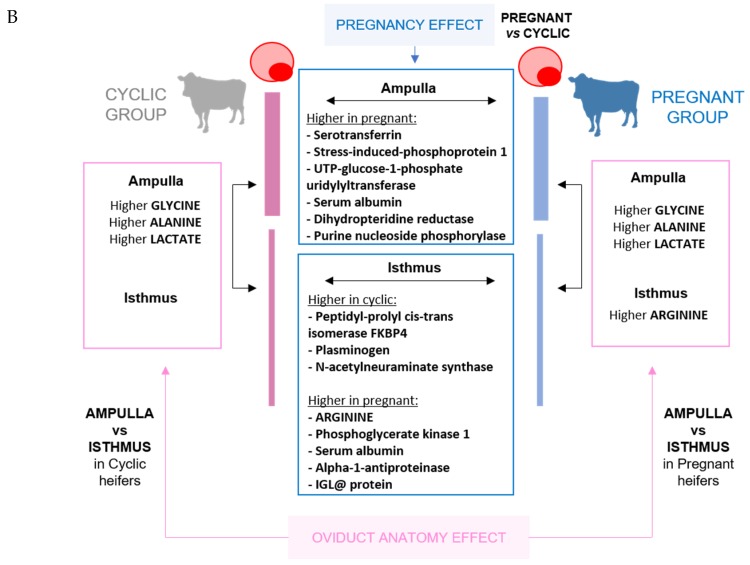
Schematic representation of the experimental design (**A**) and differences found in the oviductal fluid content in terms of proteins, amino acids and carbohydrates due to location within the oviduct or the presence of an embryo (**B**). Abbreviations: Gonadotropin-Releasing Hormone (GnRH), Controlled Internal Drug Release (CIDR), prostaglandin (PG), corpus luteum (CL).

Recovered unfertilized oocytes (cyclic group, *n* = 6) and embryos (pregnant group, *n* = 8) were consistently located in the isthmus ipsilateral to the CL on Day 3. Ampullary and isthmic flushes of the ipsilateral oviduct from 5 cyclic and 5 pregnant (in which the presence of an 8-cell embryo was confirmed) heifers were used for amino acid and carbohydrate analyses, as was a PBS blank (technical negative control) (Figure 1). Similarly, ampullary and isthmic ipsilateral oviduct flushes from 2 cyclic and 2 pregnant heifers were used for proteomic analysis, as was a PBS blank. Samples from the 3 pregnant and 3 cyclic animals were excluded due to inadequate protein recovery.

### 2.2. Proteomic Analysis

**Figure 2:**
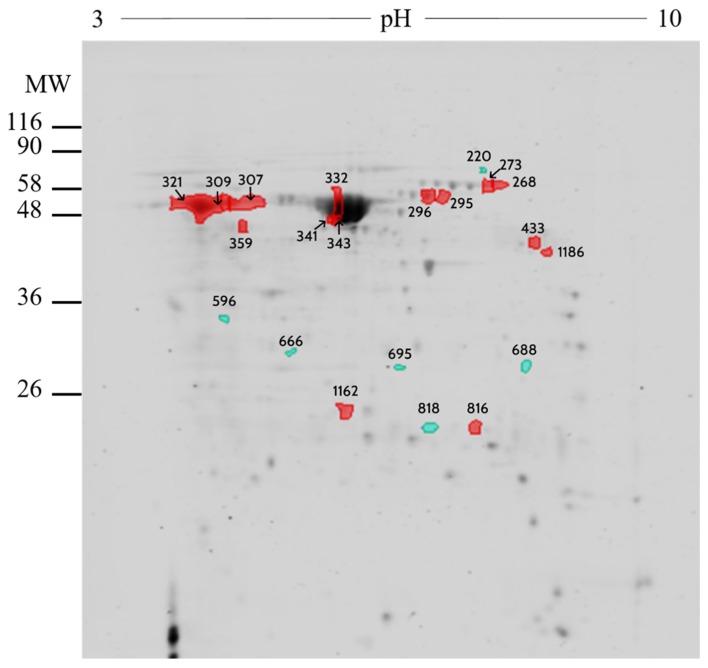
2D gel electrophoresis comparing oviduct fluid from ampulla between pregnant vs. cyclic heifers. Resolution of 150 µg of oviduct fluid in Maxi gel with non-linear gradient strips of pH 3–10 stained with Coomassie Blue. Image analysis was performed with SameSpots v.4.0 software. Spots marked in green correspond to the proteins more abundant in pregnant heifers, while spots marked in red were more abundant in cyclic heifers. MW: molecular weight.

Two-dimensional differential gel electrophoresis (2D-DiGE). 2D-DiGE analysis was performed on ampulla as well as isthmus OF between pregnant (*n* = 2) and cyclic heifers (*n* = 2). The comparative analysis of ampulla OF identified 690 protein spots, of which 21 differed (*p* < 0.02 and fold difference >1.0) in pregnant vs. cyclic heifers (Table S1). Most of these proteins (*n* = 15) were up-regulated in pregnant heifers, whereas 6 were up-regulated in cyclic heifers. None of the spots from cyclic heifers were identified by MS/MS analysis, while, 11 spots from pregnant heifers were identified by MS/MS corresponding to 6 different proteins: Serotransferrin (Q29443), Stress-induced-phosphoprotein 1 (A0A3Q1LW78), UTP-glucose-1-phosphate uridylyltransferase (A0A3Q1M010), Serum albumin (P02769), Dihydropteridine reductase (Q3T0Z7), and Purine nucleoside phosphorylase (A0A452DJA8) (Figure 2 and Table 1).

**Figure 3:**
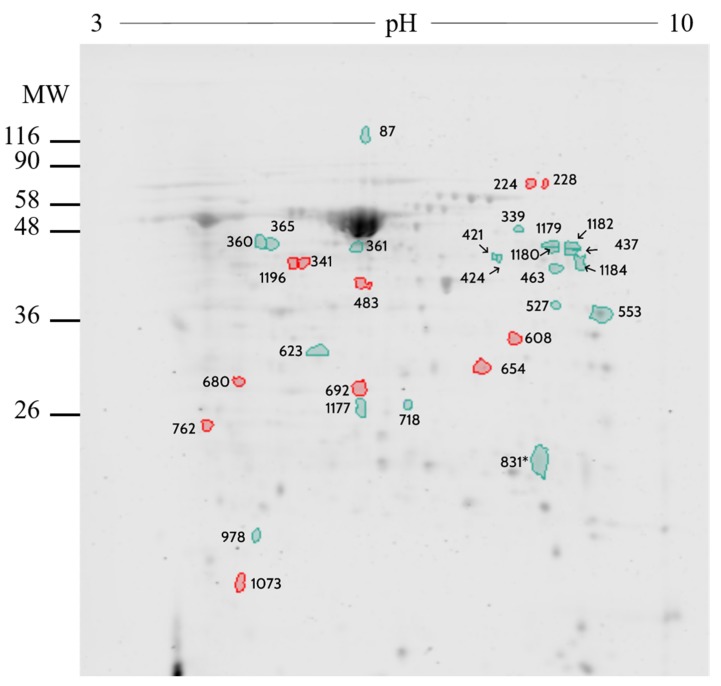
2D gel electrophoresis comparing oviduct fluid from isthmus between pregnant vs. cyclic heifers. Resolution of 150 µg of oviduct fluid in Maxi gel with non-linear gradient strips of pH 3–10 stained with Coomassie Blue. Image analysis was performed with SameSpots v.4.0 software. Spots marked in green correspond to the proteins more abundant in pregnant heifers, while spots marked in red were more abundant in cyclic heifers. MW: molecular weight.

The comparative analysis of isthmus OF identified 690 protein spots, of which 31 differed (*p* < 0.02 and fold difference >1.0) between pregnant vs. cyclic heifers (Table S2). Most of these proteins (*n* = 20) were up-regulated in pregnant heifers, whereas 11 were up-regulated in cyclic heifers. Specifically, a total of 9 spots were identified by MS/MS analysis, while 5 corresponding to 4 proteins for pregnant heifers: Phosphoglycerate kinase 1 (Q3T0P6), serum albumin (P02769), α-1-antiproteinase (P34955), and IGL@ protein (Q3T101) and 4 spots were corresponding to 3 proteins for cyclic heifers: Peptidyl-prolyl *cis*-*trans* isomerase FKBP4 (Q9TRY0), Plasminogen (P06868) and *N*-acetylneuraminate synthase (Q1RMX7) (Figure 3 and Table 2).

**Figure 4:**
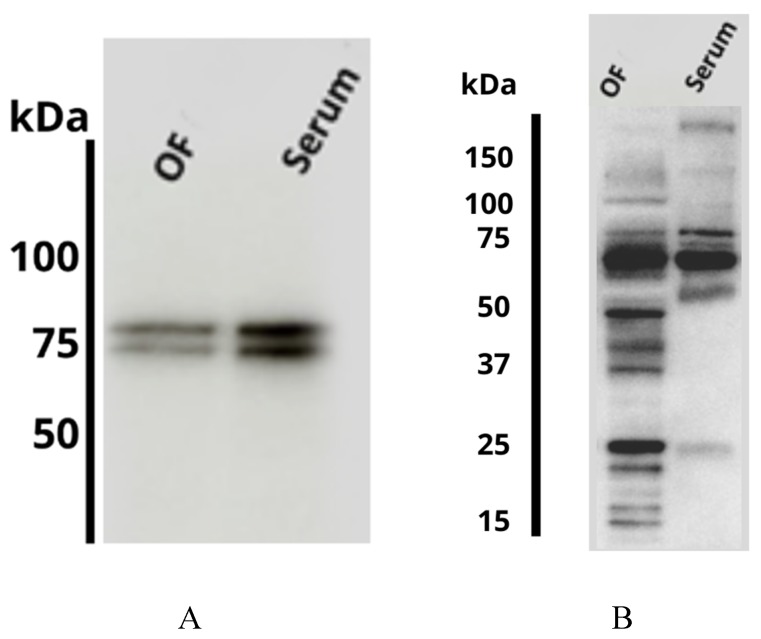
Immunoblot using (**A**) a polyclonal antibody anti-serotransferrin (1:2000) or (**B**) a polyclonal antibody α-1-antiproteinase (1:5000) in bovine oviductal fluid (OF; 0.31 µg) and serum (0.30 µg).

*Immunoblotting.* Two specific bands of approximately 75 kDa were detected in OF (from heifers slaughtered in the early luteal phase: Days 1 to 4 of the estrous cycle) using the anti-serotransferrin polyclonal antibody (Figure 4A). A similar result was obtained using the bovine serum sample (Figure 4A). In the case of α-1-antiproteinase a major band of approximately 60 kDa was detected in OF as well as in serum. Additionally, several isoform bands with different molecular weight were detected in the OF (Figure 4B). The presence of different isoforms of α-1-antiproteinase in the OF could be due to the presence of proteases in this fluid. Supporting our findings, a previous study have described the presence of α-1-antiproteinase in other biological fluids such as the cerebrospinal fluid in humans [23].

### 2.3. Amino Acid Analysis

**Figure 5:**
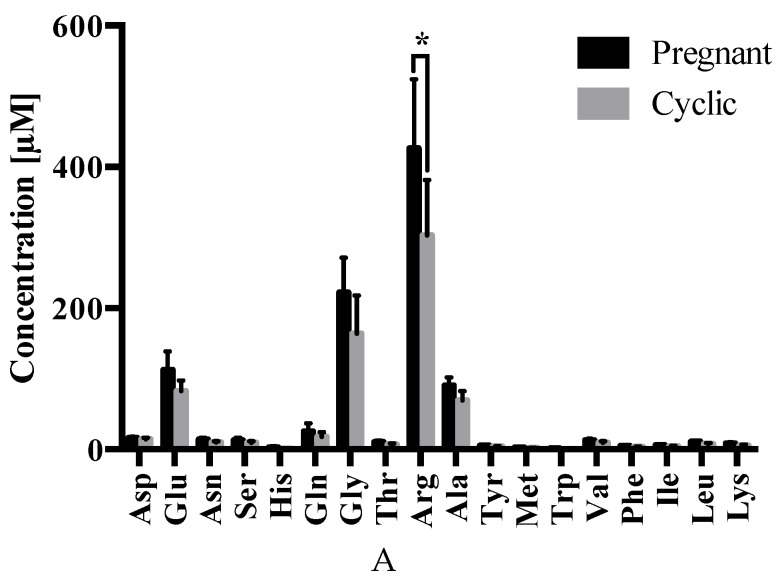

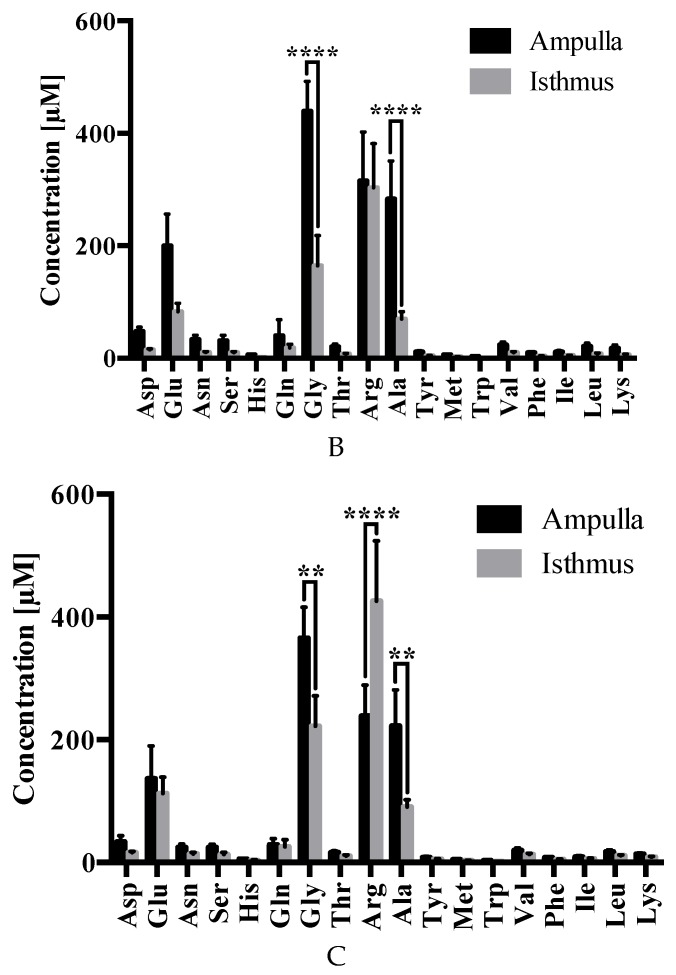
Amino acid concentrations (± SEM) in the (**A**) isthmus of cyclic vs. pregnant heifers, in addition to ampullary vs. isthmic comparisons from (**B**) cyclic and (**C**) pregnant heifers–wherein **** *p* ≤ 0.0001; ** *p* ≤ 0.01 and * *p* ≤ 0.05. Abbreviations: Aspartate (Asp), Glutamate (Glu), Asparagine (Asn), Serine (Ser), Histidine (His), Glutamine (Gln), Glycine (Gly), Threonine (Thr), Arginine (Arg), Alanine (Ala), Tyrosine (Tyr), Methionine (Met), Tryptophan (Trp), Valine (Val), Phenylalanine (Phe), Isoleucine (Ile), Leucine (Leu), and Lysine (Lys).

Regarding differences in OF composition due to the presence of an early embryo, arginine was less abundant (*p* ≤ 0.05) in the isthmus of cyclic (302.8 ± 78.5 µM) vs. pregnant (425.8 ± 98.2 µM) heifers (Figure 5A). No differences were observed in the abundance of the remaining amino acids.

With respect to differences due to oviduct anatomy, glycine and alanine were less abundant in the isthmus vs. ampulla of cyclic (163.7 ± 53.9 µM vs. 438.6 ± 53.0 µM; *p* ≤ 0.0001 and 69.0 ± 13.5 µM vs. 282.5 ± 67.8 µM; *p* ≤ 0.0001, respectively) (Figure 5B) and pregnant (221.6 ± 49.7 µM vs. 365.4 ± 50.6 µM; *p* ≤ 0.01 and 90.2 ± 11.6 µM vs. 222.1 ± 58.4 µM; *p* ≤ 0.01, respectively) (Figure 5C) heifers. Moreover, arginine was elevated (*p* ≤ 0.0001) in the isthmus (425.8 ± 98.2 µM) vs. ampulla (238.1 ± 50.5 µM) during pregnancy (Figure 5C).

### 2.4. Carbohydrate Analysis

No differences were observed in the abundance of glucose, lactate, or pyruvate in OF composition due to the presence of an embryo.

**Figure 6:**
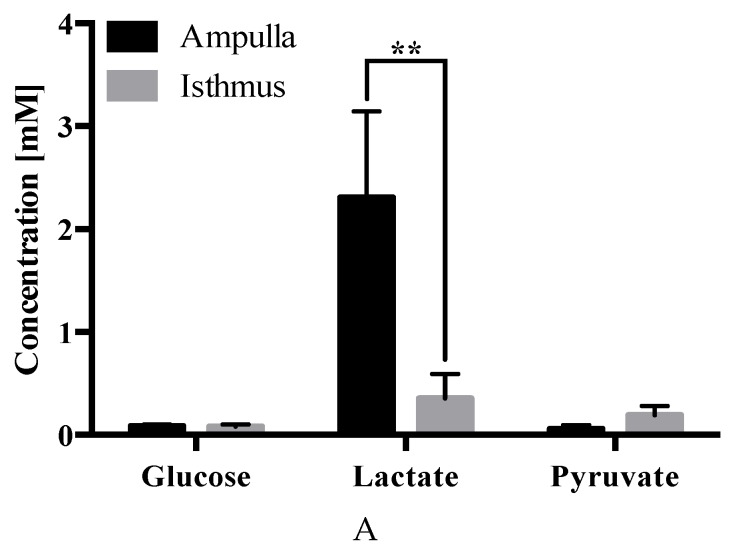

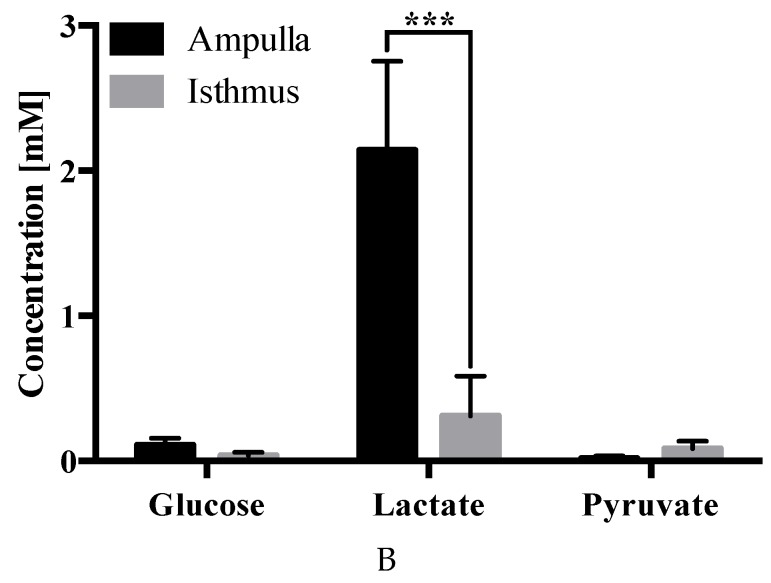
Carbohydrate concentrations (± SEM) in the ampullary vs. isthmic comparisons from (**A**) cyclic and (**B**) pregnant heifers–wherein *** *p* ≤ 0.001 and ** *p* ≤ 0.01.

In terms of differences due to the oviduct anatomy, lactate was less abundant in the isthmus vs. ampulla in both cyclic (0.4 ± 0.2 µM vs. 2.3 ± 0.8 µM; *p* ≤ 0.01) (Figure 6A) and pregnant (0.3 ± 0.3 µM vs. 2.1 ± 0.6 µM; *p* ≤ 0.001) (Figure 6B) heifers.

## 3. Discussion

The main findings of this study are that, on Day 3 post-estrus, OF composition varied based on (І) anatomical region, where isthmic metabolites were present in lower (i.e., lactate, glycine, and alanine) or higher (i.e., arginine) concentrations compared to the ampulla; and (ІІ) embryo presence, which is correlated with modifications in the oviduct proteome. Furthermore, changes in OF proteomic, amino acid, and carbohydrate composition, induced by the bovine embryo around Day 3 are presented for the first time, to our knowledge, therein providing valuable information regarding the specific needs of the developing embryo in vivo. The oviduct is a highly specialized organ able to meet the requirements of gametes and the early embryo, by adapting its luminal micro-environment in accordance with the reproductive events taking place within it [12]. Given that bovine early embryo development occurs in the isthmus until embryo migration into the uterus around Day 4, at the 16-cell stage [1], an improved knowledge of oviduct (isthmic) fluid composition and regulation during the window of early embryo development is of great importance to our understanding of fundamental developmental events.

Comparative analysis of ampullary OF proteome between pregnant vs. cyclic heifers resulted in the identification of twenty-one different spots. From these, 15 corresponded to the ampulla of pregnant and 6 to cyclic heifers. It was not possible to identify any of the 6 spots in cyclic heifers due to the low protein content present in the samples, while 6 proteins were identified by MS/MS analysis in 11 different spots of pregnant heifers (serum albumin (P02769), serotransferrin (Q29443), stress-induced-phosphoprotein 1 (A0A3Q1LW78), UTP-glucose-1-phosphate uridylyltransferase (A0A3Q1M010), dihydropteridine reductase (Q3T0Z7) and purine nucleoside phosphorylase (A0A452DJA8)).

The proteins serum albumin and serotransferrin from the blood serum stand out due to their elevated presence. Consistent with these results, it has been shown that the equine embryo is able to modulate the OF proteome by increasing the concentration of certain proteins, presumably for embryo–maternal communication [16]. Interestingly, serum albumin and serotransferrin were identified in pregnant equine OF, suggesting a possible conserved mechanism between species [16]. Moreover, the OF proteins elevated during pregnancy are present in plasma [24], suggesting that the embryo may be able to activate pathways for increasing the vascular endothelial or oviductal epithelium permeability in the oviduct, as has been observed in the presence of the embryo in the uterus of rodents just before implantation [25].

Regarding the two most abundant identified proteins in pregnant vs. cyclic ipsilateral ampulla and isthmus, serum albumin has been previously observed as one of the most abundant proteins in OF [26], despite not being expressed by oviduct epithelia [18] or by BOECs [27]. Serum albumin may aid fertilization and early embryo development by regulating OF osmolarity [28]; a physicochemical property to which gametes and embryos are sensitive [28,29]. Furthermore, in vitro studies have shown that increased BSA concentrations from 3 to 16 mg/mL accelerates embryo development [30]. Moreover, BSA addition to culture media (SOF-BSA) increases bovine embryo cell number, relative to those derived from the sheep oviduct or in vivo production [31]. In vitro embryo culture has been associated with increased incidence of large offspring syndrome (LOS) [31]; however, embryos cultured with BSA instead of serum, resulted in reduced frequency of LOS manifestation [32,33].

Serotransferrin was greater in abundance in pregnant vs. cyclic ampullary OF. Dynamic serotransferrin levels in bovine OF have been previously reported; it is more abundant in the ipsilateral (vs. contralateral) oviduct post-ovulation, and is also more abundant post-ovulation (vs. pre-ovulation) [34]. This finding, coupled with increased OF serotransferrin during pregnancy (Figure 2), suggests a role for this protein during early embryo development. Consistent with this, is the observation that transferrin receptor C (TFRC) is expressed by the pre-implantation bovine embryo, with a peak expression during the 8-cell to morula stage [35]. Serotransferrin is an iron binding transport protein that can sequester free iron, thus preventing highly toxic radical formation [36]. In vitro studies have shown that serotransferrin helps the murine embryo overcome the 2-cell block [37], and that its addition to culture media is crucial for supporting individual bovine embryo development until Day 7 [36]. Moreover, serotransferrin supplementation, in combination with other components, such as insulin and selenium, improves bovine blastocyst development rates [36].

Stress-induced phosphoprotein 1 (STIP1) was identified in the OF in the ampulla of the pregnant heifers. It has been shown to acts as a co-chaperone of HSPs 70/90 [38,39]. The presence of STIP 1 has previously been reported in the transcriptome of extracellular vesicles of the bovine oviduct; however, the protein was not detected [40]. In our study, the protein was identified by proteomic analysis in the OF of pregnant but not cyclic heifers, which may suggest that the physiological changes associated with pregnancy might lead to increased synthesis of this protein. In contrast, a transcriptomic study from our group between oviductal cells from pregnant and cyclic heifers showed that the STIP1 transcript was down-regulated in pregnant heifers [18]. This could be due to an apparent decoupling between the different processes that are required to synthesize proteins. Abundant proteins are assumed to exhibit high rates of translation or transcription. However, such proteins may be slowly translated, but can be very stable and consequently present at high final concentrations [19]. A proteomic comparative study in bovine morulae and blastocysts found that the STIP1 protein decreases in abundance in blastocysts [41], suggesting the importance of STIP1 in the early stages of embryonic development. A study in mice [42], demonstrated also the importance of STIP1 in embryo survival. Knockout mice lacking STIP1 affected the survival of embryos before implantation. The authors did not observe the expression of RNA (E10.5) or protein in the *STIP1^−/−^* mice; however, in the crosses with *STIP1^−/+^* immunoreactivity was observed in the embryo. This suggests that the STIP1 protein is acquired from the heterozygous mother. However, the mechanism by which it is acquired by the embryo is unknown. As for the bovine, it could have a similar role in embryonic development, having been detected in the OF (this study) and extracellular vesicles of the oviduct as mRNA [40]. The detection of STIP1 in the OF of pregnant heifers suggest that the embryo can acquire the STIP1 protein that is present in the OF. This process is probably mediated by extracellular vesicles according to the evidence obtained in mice where the use of recombinant protein has no effect on the embryo [42] and the presence of mRNA in the extracellular vesicles in bovine [40]. Future studies are required to clarify the transfer mechanism of the STIP1 to the embryo in the early stages of development and its physiological relevance.

The comparative analysis of isthmus OF proteome between pregnant vs. cyclic heifers resulted in the identification of 31 differentially expressed spots. From these, 20 and 11 were upregulated in pregnant and cyclic heifers, respectively, corresponding to 4 proteins (serum albumin (P02769), IGL@ protein (Q3T101), phosphoglycerate kinase (Q3T0P6) and α-1-antiproteinase (P34955)) in pregnant and 3 proteins (peptidyl-prolyl cis-trans isomerase FKBP4 (Q9TRY0), *N*-acetylneuraminate synthase (Q1RMX7) and plasminogen (P06868)) in cyclic heifers.

A protein elevated during pregnancy in the isthmus of the bovine oviduct was α-1-antiproteinase, belonging to the serine protease inhibitor family. The relevance of the presence of this protease inhibitor resides in the fact that elevated protease activity causes embryo lysis, as seen in the conditional oviduct estrogen receptor α knockout mouse [43]. Thus, the homeostasis of proteases and their inhibitors in OF is crucial as it can affect embryo viability. Therefore, it is not surprising that the α-1-antiproteinase transcript has been identified in the oviductal cells [17], presumably secreted by the oviductal epithelium. Corroborating our results, Pillai et al., [44] identified α-1-antiproteinase and other serpins (A3-1, A3-7, A3-8, D1 and H1) in bovine OF, as well as Fernandes et al., [45], in the periovulatory oviduct and uterus of goats [44], and these genes are expressed by the porcine oviduct [46].

PGK1, a non-secreted enzyme, was found in a higher concentration in the OF of pregnant heifers. This could be due to a PGK1 release into the OF through nonlethal oncotic pores, a primary secretion mechanism for several cytosolic proteins [47]. Phosphoglycerate kinase 1 (PGK1) is known as an important ATP generating enzyme in the glycolytic pathway, where it catalyzes the conversion of 1,3-diphosphoglycerate to 3-phosphoglycerate [48]. This pathway is known to be important in the production of ATP in the early stages of embryo development in cattle, however precisely why this glycolytic enzyme is present on OF is puzzling and requires further research.

Peptidyl-prolyl *cis*-*trans* isomerase FKBP4 (also known as FKBP52) protein, has been characterized as a co-chaperone for steroid hormone nuclear receptors which binds to the progesterone receptor enhancing progesterone signaling (Peattie, 1992). Studies in mice lacking the *Fkbp4* gene have compromised progesterone receptor functions leading to a failure of embryo implantation in the uterus [49,50,51]. Similarly, Demetriou et al., [52] suggested a prospective crucial role for this gene in the maintenance of pregnancy in humans. Recently, the importance of the role of FKBP4 protein was showed also in 10-day old horse embryo as it was found within the blastocoel fluid [53]. However, this study could not confirm that this protein was actively secreted into the uterine lumen, or was capable of interaction with the mare’s endometrium [53]. Although our results showed an up regulation of FKBP4 protein in cyclic heifers, further studies are needed to determine its functions in bovine pregnancy and early embryo development in the oviduct.

Plasminogen is an extracellular proenzyme that is abundant in blood plasma and most extracellular fluids [54], especially oviductal fluid from bovine and porcine [55]. In the porcine oviductal fluid plasminogen activity was found much higher in the post-ovulatory period than in the pre-ovulatory period indicating its importance during fertilization or early embryo development [56]. This was confirmed in porcine as plasminogen regulated sperm entry into the oocyte [57]. However, it did not affect embryo development in vitro in bovine [58]. Our results showed plasminogen up-regulation in isthmus of cyclic heifers compared to pregnant, which may be explained in the presence of an unfertilized oocyte and not an early embryo.

Based in two global transcriptomic data sets we recently published [18] of which in the first experiment oviduct epithelial gene expression in pregnant and cyclic heifers on Day 3 in the presence of a single embryo (pregnant) or oocyte (cyclic) was examined using Affymetrix microarrays, no differences were detected, possibly because any effect at this stage is likely to be highly local in nature given the small size of the embryo. However, amongst the genes detected, although not differentially expressed were the genes encoding four of the proteins upregulated in pregnant and cyclic heifers in the present study (*FKBP4, NANS, SERPINA1,* and *UGP2*). In the second experiment of the same study (18), in which 50 embryos were transferred to the oviducts of heifers and compared the impact of this ‘amplified’ signal to control cyclic heifers, an RNAseq analysis of isthmic epithelial cells revealed 278 differentially expressed genes, of which 123 were upregulated and 155 were downregulated in pregnant heifers [18]. While not differentially expressed, the genes coding for five out of the seven proteins upregulated in pregnant vs. cyclic heifers in the present study were detected (*FKBP4, NANS, PGK1, PLG,* and *SERPINA1*).

It is important to acknowledge that the sample size for proteomic analysis is low (due to low protein yields in 3 out of 5 animals). Thus, this component of the study should be considered as a pilot investigation and warrant further study. Nonetheless, we believe that the results are interesting and provide new information on proteins in the OF on Day 3 related with pregnancy in cattle.

The early embryo in the oviduct is exposed to an OF that undergoes temporal changes in its ionic and biochemical composition. Ionic composition and potassium levels are higher near estrus [3], while the concentrations of sodium and calcium decrease across days, being higher on Day 0 than on Days 2, 4, and 6 of the oestrous cycle [59]. Comparisons of metabolite concentrations of OF from oviduct ipsilateral to the corpus luteum between the postovulatory (Days 1–5) and the early to mid-luteal (Days 6–12) phase showed differences of creatine, glucose-1-phosphate and histamine [11].

Regarding amino acids, our results showed glycine and alanine less abundant in the isthmus vs. ampulla of pregnant (Figure 5C) and cyclic (Figure 5B) heifers, whereas arginine was elevated in the isthmus vs. ampulla of pregnant heifers. These functional anatomical differences support previous findings regarding the oviduct transcriptome, in which 2287 differentially expressed genes were found between the isthmus and the ampulla of the oviduct ipsilateral to the CL in pregnant heifers [17]. Amino acids are required for normal embryo development and act as energy sources, osmolytes, pH regulators, signaling molecules, and heavy metal chelators, in addition to being central to protein and nucleic acid synthesis [60,61]. In vitro studies have shown that bovine pre-implantation embryo amino acid depletion and production rates are indicative of developmental competency [62].

Glycine and alanine, two of the amino acids whose concentrations fluctuated in the present study, have been previously observed to be the two most abundant amino acids in bovine OF, with glycine as the predominant one [3,60]. Glycine is an organic intracellular osmolyte, protecting embryos from extracellular ionic and osmotic pressures [29,63,64,65]. Additional roles include serving as an energy source and precursor for proteins, purines, and pyrimidines [8]. Alanine exhibits pH modulating properties and may too protect the embryo from osmotic stress [60]. Alanine is presumed to be the end-product of a metabolic pathway that reduces embryotoxic ammonia accumulation [60,66,67]. Meanwhile some authors suggest that embryos secrete significant alanine to sequester ammonium ions [68], others consider that alanine does not play a major role in early embryo development given that it is not consumed, but rather is secreted, by the embryo [69]. However, in vitro studies show improved bovine embryo development following glycine and alanine supplementation [70,71]. In the present study, glycine and alanine concentrations were lower in the isthmus vs. ampulla, irrespective of pregnancy, which could reflect the smaller mucosal surface area of the isthmus vs. ampulla [4].

Arginine, which also showed anatomical variations (Figure 5C), was higher in the pregnant vs. cyclic isthmus (Figure 5A). This may arise due to changes in hormone profile or response to an embryo-derived factor. In vitro studies have shown that the bovine embryo begins to consume substantial arginine at the 8-cell stage [72], and that arginine addition to culture medium improve blastocyst formation and hatching [73]. In vivo, dietary arginine supplementation improves porcine reproductive performance [74], and in the bovine, porcine, and ovine, arginine has been identified as a key player in peri-elongation embryo development [75,76,77,78]. These results, coupled with the increased arginine observed in the isthmus—but not the ampulla—of pregnant vs. cyclic heifers (Figure 5A), suggest an important role for arginine in early bovine embryo development. Additionally, in relation to transporters, in the transcriptomic study mentioned above [18] we identified 5 solute carriers (*SLC1A4, SLC18A2, SLC24A4, SLC26A3,* and *SLC16A11*) which were differentially expressed in the isthmus of pregnant vs. cyclic heifers, including an amino acid transporter, a sodium/potassium/calcium exchanger and a chloride anion exchanger.

Regarding carbohydrates, lactate levels differed between ampulla and isthmus; lactate was present in lower concentrations in the isthmus vs. ampulla of cyclic (Figure 6A) and pregnant (Figure 6B) heifers. Carbohydrates (such as glucose, pyruvate, sucrose and lactate) in OF serve to nourish the oocyte, sperm, and early embryo [3]. The early in vitro embryo uses oxidative metabolism to generate energy during the first stages of development, primarily using pyruvate and lactate as a source, whereas glycolytic activity increases as the embryo develops [11]. Regarding lactate, an in vivo metabolomic profiling of the bovine OF showed that it is the most abundant energy substrate throughout the estrous cycle [11]. Lactate is largely produced and secreted by the oviduct epithelium, as inferred by bovine OF lactate levels being 5–6-fold higher than those of plasma [79], an observation consistent with [80], who showed, in the rabbit, that 75% of lactate is produced by the tubal epithelial cells from vascular glucose, whereas 25% filters from the blood. Moreover, the rabbit ampulla comprises 1.8 times more lactate than the isthmus [4], in line with our results in the bovine. Furthermore, it has been proposed in the mouse that lactate, an acidic carbohydrate produced by the blastocyst, plays a role in (І) disaggregating uterine epithelia for trophoblast invasion facilitation, (ІІ) signals VEGF recruitment to promote angiogenesis, and (ІІІ) promotes local immune tolerance by forming a pH gradient [81]. Investigating these phenomena in the context of the bovine oviduct are areas for further work, particularly in lieu of the observation that lactate reduction was more pronounced in the isthmus of pregnant (*p* = 0.0003) vs. cyclic (*p* = 0.0023) heifers (Figure 6).

## 4. Material and Methods

All experimental procedures involving animals were approved by the Animal Research Ethics Committee of University College Dublin and were licensed by the Health Products Regulatory Authority, Ireland, in accordance with Statutory Instrument No. 543 of 2012 under Directive 2010/63/EU on the Protection of Animals used for Scientific Purposes.

### 4.1. Animal Model

Throughout the experiment, all animals were housed indoors on a slatted floor and were fed a diet consisting of grass and maize silage supplemented with a standard beef ration. The oestrous cycles of crossbred beef heifers (*n* = 19, predominantly Charolais and Limousin cross; aged 23.0 ± 0.7 (± s.e.m.) months and weighing 583.26 ± 12.5 kg (± s.e.m.)) were synchronized using a 7-day Controlled Internal Drug Release (CIDR; 1.38 g progesterone; Pfizer) insert combined with a dose of 0.02 mg of a Gonadotropin-Releasing Hormone (GnRH) agonist (Buserelin; Receptal; Intervet, Dublin, Ireland) and an administration of 15 mg Prostaglandin F2α analog (Prosolvin; Intervet) the day before CIDR removal. To detect signs of estrus, heifers were observed four times per day commencing 30 h after CIDR withdrawal, and only those recorded in standing estrus (Day 0) were used. Heifers were randomly allocated to one of two groups: (*a*) non-bred (i.e., cyclic) or (*b*) pregnant, which were artificially inseminated 12 and 24 h after the first sign of estrus, with frozen-thawed semen from a bull of proven fertility. The experimental design is illustrated in Figure 1.

### 4.2. Sample Collection

OF for proteomic, amino acid and carbohydrate analysis. On Day 3 post-estrus, animals were slaughtered in a commercial abattoir. The reproductive tract was removed, sealed in a plastic bag, transported to the laboratory on ice, and processed within 3.5 h after slaughter. Oviducts ipsilateral to the corpus luteum (CL) were trimmed free of tissue, and, after removal of the infundibulum and the utero–tubal junction, the oviduct was divided into two parts at the ampullary–isthmic junction, defined by the point at which the oviduct visibly narrows [17]. Each ampulla and isthmus was individually flushed with 500 μL protein-free PBS, and the presence and location of an unfertilized oocyte or an embryo was verified under a microscope. The 500 μL flush from each section was centrifuged for 1 min at 930× *g*. The supernatant was recovered and stored at −80 °C until proteomic, amino acid and carbohydrate analysis.

OF for protein immunoblotting. OF from oviducts ipsilateral to the CL were obtained from heifers slaughtered in the early luteal phase (Days 1 to 4 of the estrous cycle), as determined by ovarian morphology according to [82]. The oviducts were transported to the laboratory in a sealed plastic bag on ice, and processed within 3.5 h within after slaughter. OF was collected as described by [83] with minor modifications. Briefly, the surrounding fat and connective tissues were carefully removed, and each oviduct was washed three times in PBS without Ca^2+^/Mg^2+^. Then, under sterile conditions, OF was collected by gentle squeezing from the isthmus to ampulla. OF was centrifuged twice at 7000× *g* for 10 min at 4 °C to remove cellular debris. The resulting supernatant was aliquoted and stored at −80 °C until use. Serum was isolated from peripheral circulation via the ear pinnacle from heifers post-slaughter in the early luteal phase (Days 1 to 4 of the estrous cycle), as determined by ovarian morphology according to [82], in Primavette^®^ serum tubes. Blood samples were subsequently centrifuged at 2000× *g* for 10 min at 4 °C, transferred to new vials, and stored at −80 °C until analysis.

### 4.3. Proteomic Analysis

All Day 3 post-estrus OF samples from isthmus and ampulla (cyclic and pregnant heifers) were passed through a 0.5 mL Microcon-10 kDa Centrifugal Filter with Ultracel-10 NMWL membrane to concentrate the protein in the retained fraction for proteomic analysis; the fraction that passed through the filter was stored at −80 °C until use for amino acid and carbohydrate analysis.

Total protein concentration was determined by the bicinchoninic acid (BCA) method, in accordance with the manufacturer’s instructions (Pierce BCA Protein Assay Kit, Thermo Scientific, Rockford, IL, USA).

Two-dimensional differential gel electrophoresis (2D-DiGE). Total protein (150 µg) from the ipsilateral ampulla and isthmus from 2 cyclic and 2 pregnant heifers was used; samples yielding a lower amount of protein did not proceed with analysis. Samples were labelled with CyDyes following the manufacturer’s instructions (Amersham CyDye DIGE Fluors (minimal dyes, 5 nmol) for Ettan DIGE (GE Healthcare, 25-8010-65)). Briefly, CyDyes were reconstituted in anhydrous dimethylformamide, and samples were diluted in labeling buffer (30 mM Tris pH 8.5, 7 M urea, 2 M tiourea, 4% CHAPS) and incubated with CyDyes for 30 min on ice, and in darkness, at a ratio of 400 pmol CyDye/100 µg protein. CyDye3 and CyDye5 were used to label individual samples from cyclic or pregnant samples, while CyDy2 was used for labelling a mixture of both samples as an internal standard. Ten micromoles of lysine was used for stopping the labelling. Samples were subsequently pooled and mixed with isoelectrofocusing buffer (8 M urea, 2% CHAPS, 50 mM DTT, 0.2% (*w*/*v*) Bio-Lyte^®^ 3/10 ampholytes, and bromophenol blue (trace)).

Isoelectric focusing (IEF) was performed using immobilized pH gradient strips (ReadyStrips IPG strips pH 3–10, 17 cm (Bio-Rad, Hercules, CA, USA)) with the Protean^®^ IEF cell system (Bio Rad). The 3-step focusing program ran as follows: 250 V for 20 min, followed by a linear increase to 10,000 V for 2.5 h, prior to 10,000 V for approximately 4 h, until reaching a total of 40,000 VH, with a maximum current of 50 mA per strip. Once the isoelectric focusing was completed, the strips were equilibrated for 10 min with a solution comprising 6 M urea, 2% SDS, 0.375 M Tris-HCl (pH 8.8), 20% glycerol, and 2% DTT. Then strips were identically equilibrated in a solution comprising 6 M urea, 2% SDS, 0.375 M Tris-HCl (pH 8.8), 20% glycerol, and 2.5% iodoacetamide. Strips were then adjusted to a custom SDS-polyacrylamide gels (12%) supplemented with agarose (0.5% low melting point agarose in 25 mM Tris, 192 mM glycine, 0.1% SDS, with a trace of bromophenol blue). The second electrophoretic step was performed using a 2-step program with 16 mA/gel for 30 min, and then 24 mA/gel for approximately 6 h.

Gels were scanned with a Typhoon 9410 (Amersham) in accordance with the manufacturer’s suggested parameters. Image analysis was performed using Progenesis SameSpots software v.4.0. The spot selection criteria were *p* < 0.02, as determined by ANOVA, and a fold difference in intensity >1.5.

Trypsin digestion and HPLC-ESI-MS/MS analysis. The analysis was performed as previously reported [84]. Briefly, spot detection was achieved by staining gels with PageBlue Coomassie blue protein staining solution, in line with the manufacturer’s instructions (Thermo Fisher Scientific) and manually cut for protein identification by mass spectrometry analysis (MS/MS). A high-performance liquid chromatography-mass spectrometry (HPLC-ESI-MS/MS) system consisting of an Agilent 1100 Series HPLC (Agilent Technologies) connected to an Agilent Ion Trap XCT Plus mass spectrometer equipped with an electrospray ionization interface was used to separate and analyze trypsinized digests.

Immunoblotting. To confirm the presence of the proteins serotransferrin and α-1-antiproteinase in bovine OF and serum, both fluids were obtained as described above. Subsequently, 0.5 µl diluted OF (0.31 µg) and serum (0.30 µg) were separated by SDS-PAGE (Novex^TM^ WedgeWell^TM^ 16% Tris-Glycine Gel, Invitrogen, Waltham, Massachusetts, USA) and transferred to PVDF membranes. Transfer conditions were 40 V for 1 h. Membranes were subsequently blocked in 1% TBTS-BSA (bovine serum albumin), overnight, at 4 °C, and probed with either an anti-serotransferrin HRP-conjugated sheep polyclonal antibody (ab112892, Abcam) at a 1:2000 dilution in 1% TBTS-BSA or anti-α 1 antiproteinase HRP-conjugated goat polyclonal antibody (ab191350, Abcam) at a 1:5000 dilution in 1% TBTS-BSA. After washing, membranes were incubated with 1 mL Pierce^®^ ECL 2 western blotting substrate (Thermo Scientific) at room temperature for 5 min. The chemiluminescent signal was acquired with ImageQuant LAS500 (GE Health Life Sciences).

### 4.4. Amino acid Analyses

The amino acid composition of OF flushes was quantitatively determined as previously reported [85]. In brief, amino acids present were derivatized with orthopthaldialdehyde reagent supplemented with 1 mg/mL β-mercaptoethanol prior to analytical separation by reverse phase high performance liquid chromatography using an Agilent 1100 system coupled with a Phenomenex HyperClone 5 mm C-18 ODS 250 mm × 4.6 mm (extended) column. A gradient elution using two buffers: (A) 80% 83 mM sodium acetate (pH 5.9) + 19.5% methanol + 0.5% tetrahydrofuran, and (B) 80% methanol + 20% 83 mM sodium acetate ran for 60 min at a flow rate of 1.3 mL/min. The column oven was set to 30 °C and 18 detectable amino acid conjugates were separated by retention time—as detected by fluorescence at 450 nm when excited at 330 nm. Identification and quantification were conducted by peak area comparisons against known standards.

### 4.5. Carbohydrate Analyses

The carbohydrate composition of OF flushes was quantitatively determined as previously reported [86]. In brief, glucose, lactate, and pyruvate concentrations were measured individually and indirectly via their enzymatic conversion to produce spectrophotometrically detectable nicotinamide by-products. Sample and standard fluorescence was detected using a FLUOstar Omega microplate reader (BMG LabTech). Samples were diluted to fit within standard curves, and quantification was conducted by comparing sample and standard curve fluorescence intensities, accounting for dilution.

### 4.6. Statistical Analyses

For proteomic assessment, the results of the image analysis were compared by ANOVA, using Progenesis SameSpots software v.4.0. For both amino acid and carbohydrate analyses, significant differences were determined by two-way analysis of variance coupled with the Holm-Sidak nonparametric *post hoc* analysis, using GraphPad Prism 6.

## 5. Conclusions

In conclusion, the presented data demonstrate that bovine OF composition is anatomically dynamic and influenced by the presence of an early embryo. On Day 3 post-estrus, in the ipsilateral isthmus, arginine was elevated, whereas glycine, alanine, and lactate were depleted, relative to the ampulla. Moreover, in the presence of an embryo, OF exhibited higher concentrations of serum albumin, serotransferrin, stress-induced phosphoprotein 1, phosphoglycerate kinase 1 and serpina1, to facilitate optimal embryo development. These findings should be confirmed on a larger sample size but nonetheless enhance our understanding of the micro-environment in which fundamental early developmental events occur and offer scope to improve embryo development and quality via better mimicking in vivo OF dynamics in vitro.

## Figures and Tables

**Table 1:** Proteins identified by MS/MS in the oviduct fluid from ampulla of pregnant vs. cyclic heifers.

Number	UniprotKB	Protein Name	Symbol	Fold
309	P02769	Serum albumin	ALB	1.19
332	P02769	Serum albumin	ALB	1.27
341	P02769	Serum albumin	ALB	1.41
343	P02769	Serum albumin	ALB	1.21
1162	A0A452DJA8	Purine nucleoside phosphorylase	PNP	1.03
816	Q3T0Z7	Dihydropteridine reductase	QDPR	1.11
296	A0A3Q1LW78	Stress-induced-phosphoprotein 1	STIP1	1.98
268	Q29443	Serotransferrin	TF	2.27
273	Q29443	Serotransferrin	TF	2.07
295	Q29443	Serotransferrin	TF	1.42
433	A0A3Q1M010	UTP--glucose-1-phosphate uridylyltransferase	UGP2	1.54

**Table 2:** Proteins identified by MS/MS in the oviduct fluid from isthmus of pregnant vs. cyclic heifers.

Number	UniprotKB	Protein Name	Symbol	Fold
437	P02769	Serum albumin	ALB	1.08
831	Q9TRY0	Peptidyl-prolyl cis-trans isomerase FKBP4	FKBP4	1.24
1179	-	-	IGHG	1.49
463	-	-	IGHG	1.28
339	-	-	IGHG	1.27
978	Q3T101	IGL@ protein	IGL	1.59
527	Q1RMX7	*N*-acetylneuraminate synthase	NANS	1.12
718	Q3T0P6	Phosphoglycerate kinase 1	PGK1	1.00
360	P06868	Plasminogen	PLG	1.16
1182	P06868	Plasminogen	PLG	1.08
1180	P34955	α-1-antiproteinase	SERPINA1	1.33
1184	P34955	α-1-antiproteinase	SERPINA1	1.23

## Supplementary Materials

The following are available online at https://www.mdpi.com/1422-0067/21/5/1681/s1, Table S1. Differentially expressed protein spots (*n* = 21; *p* < 0.02 and fold difference >1.0) in the oviductal fluid from ampulla of pregnant vs. cyclic heifers. Table S2. Differentially expressed protein spots (*n* = 31; *p* < 0.02 and fold difference >1.0) in the oviductal fluid from isthmus of pregnant vs. cyclic heifers.
